# Treatment sequence with gilteritinib and allogeneic haematopoietic stem cell transplantation in relapsed/refractory FMS‐like tyrosine kinase 3 (FLT3)‐mutated acute myeloid leukaemia patients: A multicentre real‐world study

**DOI:** 10.1111/bjh.70735

**Published:** 2026-08-18

**Authors:** Desiree Kunadt, Sven Zukunft, Christoph Röllig, Stephan Metzelder, Andreas Burchert, Eva Wagner‐Drouet, Oliver Kriege, Christoph Schliemann, Matthias Stelljes, Julian Ronnacker, Wolfgang Bethge, Jan Frederic Weller, Björn Steffen, Gesine Bug, Khouloud Kouidri, Claudia Baldus, Lars Fransecky, Elisa Sala, Uwe Platzbecker, Klaus Metzeler, Sabine Kayser, Inken Hilgendorf, Andreas Hochhaus, Tim H. Brümmendorf, Edgar Jost, Anna Dorottya Doleschall, Thomas Schroeder, Jennifer Kaivers, Lambros Kordelas, Udo Holtick, Julia M. Unglaub, Tim Sauer, Paul Sebastian Jäger, Guido Kobbe, Elena Stauffer, Johanna Tischer, Tobias Herold, Normann Steiner, David Nachbaur, Nicolaus Kröger, Jochen J. Frietsch, Sabrina Kraus, Hermann Einsele, Mathias Hänel, Judith Schaffrath, William Krüger, Anne Brandenburg, Tobias A. W. Holderried, Laura Reinhardt, Markus Maulhardt, Christian Thiede, Martin Bornhäuser, Johannes Schetelig, Jan M. Middeke, Friedrich Stölzel, for the German Cooperative Transplant Study Group and Study Alliance Leukemia (SAL)

**Affiliations:** ^1^ Medizinische Klinik und Poliklinik I, Universitätsklinikum Carl Gustav Carus, TU Dresden Dresden Germany; ^2^ Department of Internal Medicine, Hematology, Oncology and Immunology Philipps University Marburg and University Hospital Gießen and Marburg Marburg Germany; ^3^ Department of Hematology, Oncology and Pneumology University Medical Center Mainz Mainz Germany; ^4^ Medizinische Klinik A, Universitätsklinikum Münster Münster Germany; ^5^ Department for Hematology, Oncology, Clinical Immunology and Rheumatology University Hospital Tübingen Tübingen Germany; ^6^ University Hospital Eppendorf Hamburg Germany; ^7^ Medizinische Klinik 2, Hämatologie/Onkologie, Goethe‐Universität Frankfurt am Main Germany; ^8^ Klinik für Innere Medizin II, Universitätsklinikum Schleswig‐Holstein Kiel Germany; ^9^ Department of Internal Medicine III University Hospital Ulm Ulm Germany; ^10^ Department of Hematology, Cell Therapy, Hemostaseology and Infectious Diseases University Hospital Leipzig Leipzig Germany; ^11^ Medical Faculty Mannheim Institute of Transfusion Medicine and Immunology, Heidelberg University, German Red Cross Blood Service Baden‐Württemberg‐Hessen Mannheim Germany; ^12^ NCT Trial Center, National Center of Tumor Diseases, German Cancer Research Center (DKFZ) Heidelberg Germany; ^13^ Klinik für Innere Medizin II, Universitätsklinikum Jena Jena Germany; ^14^ Medizinische Klinik IV, Uniklinik RWTH Aachen Aachen Germany; ^15^ Klinik für Hämatologie, Universitätsklinikum Essen Essen Germany; ^16^ Department I of Internal Medicine Medical Faculty and University Hospital, Cologne, University of Cologne Cologne Germany; ^17^ Department of Hematology, Oncology and Rheumatology University Hospital Heidelberg Heidelberg Germany; ^18^ Department of Hematology, Oncology and Clinical Immunology University of Duesseldorf Duesseldorf Germany; ^19^ Department of Medicine III University Hospital, LMU Munich Munich Germany; ^20^ Department of Hematology, Oncology, and Stem‐Cell Transplantation Munich Hospital Bogenhausen, Munich Municipal Hospital Group Munich Germany; ^21^ Division of Hematology and Medical Oncology, Department of Internal Medicine V Medical University of Innsbruck Innsbruck Austria; ^22^ Medizinische Klinik und Poliklinik II, Universitätsklinikum Würzburg Würzburg Germany; ^23^ Medizinische Klinik III, Klinikum Chemnitz Chemnitz Germany; ^24^ Department of Hematology/Oncology Martin Luther University Halle/Wittenberg Halle Germany; ^25^ Clinic for Internal Medicine C—Haematology and Oncology, Stem Cell Transplantation and Palliative Care University Medicine Greifswald Greifswald Germany; ^26^ Medical Clinic and Polyclinic III Internal Medicine With a Focus on Oncology, Hematology, and Rheumatology, University Hospital Bonn, University of Bonn Bonn Germany; ^27^ Division of Hematology Medical University of Graz Graz Austria; ^28^ Department of Hematology, Oncology, and Stem Cell Transplantation Freiburg University Medical Center Freiburg Germany; ^29^ Department of Hematology and Medical Oncology University Medicine Göttingen Göttingen Germany; ^30^ National Center for Tumor Diseases Dresden Germany; ^31^ German Consortium for Translational Cancer Research (DKTK), DKFZ Heidelberg Germany; ^32^ Clinical Trials Unit DKMS Dresden Germany

**Keywords:** allogeneic haematopoietic stem cell transplantation, AML, FLT3, gilteritinib, maintenance, relapse, survival

## Abstract

Patients with relapsed or refractory acute myeloid leukaemia (r/r AML) harbouring FMS‐like tyrosine kinase 3 (FLT3) mutations generally have poor prognosis and limited treatment options. The second‐generation FLT3 inhibitor gilteritinib provides better disease control compared to standard relapse therapies and was approved based on two randomized trials. We analysed its efficacy and safety in a real‐world cohort of r/r AML patients undergoing gilteritinib treatment including allogeneic haematopoietic stem cell transplantation (alloHSCT). Data were collected from all consecutive r/r AML patients with FLT3‐internal tandem duplication and/or FLT3‐tyrosine kinase domain mutations receiving gilteritinib at 25 centres. Response and survival were analysed using descriptive and correlative statistics. A total of 156 patients were analysed, 55.1% with relapse after previous alloHSCT, and 46.2% with prior FLT3 inhibitor exposure. The overall complete remission/incomplete remission (CR/Cri) rate was 45.3%, with a median overall survival (OS) of 10.0 months. Patients receiving gilteritinib only had a 1‐year OS of 34%, while those with consolidating alloHSCT had a 1‐year OS of 76%–92%, depending on gilteritinib maintenance post‐transplant. Resuming gilteritinib post‐transplant led to an 85.7% CR/CRi rate. Our real‐world data confirm that gilteritinib is an effective single‐agent therapy for r/r AML with FLT3 mutations, with best outcomes when used as a bridge to transplant and post‐transplant maintenance.

## INTRODUCTION

Despite a constant improvement of first‐line therapy for fit acute myeloid leukaemia (AML) patients, a considerable number of patients experience refractory disease or relapse, the latter often accompanied by clonal genetic evolution.^1^, ^2^, ^3^, ^4^ In relapsed or refractory AML (r/r AML), curative options are limited and prognosis remains poor.^5^, ^6^, ^7^ Therefore, targeted therapies based on mutational characteristics have become a major focus of scientific and clinical interest to improve survival.

FMS‐like tyrosine kinase 3 (FLT3) receptors are expressed early in haematopoiesis.^8^ FLT3 mutations (*FLT3* ^mut^) have been identified as drivers of leukaemogenesis through constitutive receptor activation and downstream signalling, inducing proliferation of multipotent progenitor and leukaemic blasts.^9^, ^10^, ^11^, ^12^, ^13^, ^14^, ^15^ *FLT3* ^mut^ are among the most common recurrent aberrations in AML and are detected in approximately 30% of patients at initial diagnosis.^3^, ^16^ The most frequent subtypes are internal tandem duplications (*FLT3*‐ITD; 20%–25%), and point mutations in the tyrosine kinase domain (*FLT3*‐TKD; 7%–10%).^17^, ^18^, ^19^ *FLT3*‐ITD mutations have consistently been associated with poor survival and early relapse.^3^, ^20^, ^21^ In contrast, reports on the prognostic impact of *FLT3*‐TKD mutations remain inconsistent.^22^, ^23^ Several studies have demonstrated an increased frequency of *FLT3* ^mut^ at relapse, highlighting their role in clonal evolution and emphasizing the need for repeated molecular screening.^15^, ^16^, ^24^, ^25^ Approximately 75% of *FLT3* ^mut^ persist at relapse compared to initial diagnosis, while about 16% are newly acquired.^26^ Interestingly, the addition of FLT3 inhibitors (FLT3i) such as midostaurin to first‐line therapy results in loss of detectable *FLT3*‐ITD in approximately 50% of patients relapse.^27^

Outcome of *FLT3* ^mut^ r/r AML treated with conventional salvage strategies without FLT3i remains poor.^28^, ^29^, ^30^, ^31^, ^32^, ^33^, ^34^ Over the past decade, several first‐ and second‐generation FLT3i have been developed and incorporated into treatment algorithms improving survival. Next‐generation FLT3i gilteritinib demonstrated a significant clinical benefit regarding composite complete remission (CR) rates, event‐free and overall survival (OS) compared to salvage chemotherapy in *FLT3* ^mut^ r/r AML, resulting in FDA and EMA approval.^33^, ^35^, ^36^ Gilteritinib is a potent, highly selective type I FLT3i active against both *FLT3*‐ITD and *FLT3*‐TKD mutations, with additional inhibitory activity against KIT proto‐oncogene, receptor tyrosine kinase and AXL receptor tyrosine kinase, which have been implicated in mechanisms of FLT3i resistance.^37^, ^38^, ^39^ Median OS in two randomized controlled trials ranged from 9.3 months (ADMIRAL) to 9.6 months (COMMODORE).^33^, ^34^ Nevertheless, remission duration with single‐agent FLT3i is short, and there is an unmet need to define optimal sequential treatment strategies incorporating allogeneic haematopoietic stem cell transplantation (alloHSCT) and maintenance approaches.^40^, ^41^

We therefore performed a large multicentre analysis evaluating outcomes of *FLT3* ^mut^ r/r AML patients receiving gilteritinib in different therapeutic settings, including subsequent alloHSCT and/or gilteritinib maintenance, to provide robust real‐world data and further define the role of gilteritinib in the transplant context.

## PATIENTS AND METHODS

### Patients and study design

For this analysis, data from all patients treated between 2019 and 2022 at the 25 participating centres of the Study Alliance Leukemia Registry (NCT03188874) and the German Cooperative Transplant Study Group were collected. Documented variables included patient demographics, disease characteristics, treatment details, therapeutic sequences, timing of alloHSCT and outcome parameters, which were recorded using a comprehensive standardized data table completed by the participating centres. Both patients considered fit for intensive induction therapy (IT) with subsequent alloHSCT and unfit patients receiving gilteritinib were analysed within the respective therapeutic setting. Patients were treated with gilteritinib according to the EMA label from 2019 and 2022.

All patients provided written informed consent for analysis of their data. The study was approved by the respective institutional ethics committees and conducted in accordance with the Declaration of Helsinki.

### Molecular and cytogenetic analyses

Pre‐treatment samples and/or samples obtained at relapse or refractory disease from bone marrow or peripheral blood were used for genomic deoxyribonucleic acid (DNA) isolation and cytogenetic analysis. After DNA extraction, samples were screened for *FLT3*‐ITD and *FLT3*‐TKD mutations as well as additional molecular aberrations as previously described.^19^, ^42^ The lower limit of detection was 0.5% for ultradeep next‐generation sequencing (NGS) and 1%–5% for panel NGS. Cytogenetic analyses were performed according to local standards.

### Definitions and response criteria

Patients harbouring *FLT3*‐ITD and/or *FLT3*‐TKD mutations at relapse or refractory disease who qualified for salvage treatment with gilteritinib were included. Gilteritinib treatment duration had to be at least 15 days, corresponding to half an induction cycle. Disease risk was stratified according to the European LeukemiaNet (ELN) 2017 recommendations^43^ and AML subtypes were classified based on the 2016 World Health Organization classification.^44^ Treatment response and relapse were defined according to ELN2017 criteria. The haematopoietic stem cell transplantation‐specific comorbidity index (HSCT‐CI) and conditioning intensity were defined as previously described (Table S1).^45^, ^46^ Grading of non‐haematological toxicity was performed using the Common Terminology Criteria for Adverse Events. OS for the entire cohort was defined as the time from initiation of gilteritinib treatment to death from any cause. Survival analyses were performed for patients ≤75 years. For the subgroup of patients receiving gilteritinib as maintenance therapy after alloHSCT, survival was calculated from the start of maintenance treatment.

### Statistical analyses

Statistical as‐treated analyses evaluating the impact of gilteritinib in *FLT3* ^mut^ r/r AML were performed using the statistical computing environment R (Version 4.3.3). Continuous variables were compared using the Kruskal–Wallis test, while categorical variables were analysed using the chi‐square test. Effects of therapeutic sequences was assessed using Cox regression models with alloHSCT modelled as a time‐dependent covariate. Due to this time‐dependent modelling, all patients initially start in the non‐alloHSCT group. Consequently, the number at risk in the non‐alloHSCT group at the start of the observation period also includes patients who are subsequently allografted. The number at risk in the alloHSCT group at time point zero reflects the number of patients who transitioned from the non‐alloHSCT group to the alloHSCT group before the first event or censoring occurred. Kaplan–Meier estimates were generated to visualize survival according to therapeutic sequence, and log‐rank tests were applied to compare OS and progression‐free survival. Cox regression was also used to identify independent prognostic factors and to estimate univariate and adjusted hazard ratios (HRs). Multivariable models included therapeutic sequences of gilteritinib, age at diagnosis, ELN risk group, secondary AML, therapy‐related AML and Karnofsky index at start of gilteritinib. A two‐sided *p*‐value <0.05 was considered significant.

## RESULTS

### Patients' characteristics

A total of 156 patients with relapsed or primary refractory AML and polymerase chain reaction‐based molecular detection of either *FLT3*‐ITD and/or *FLT3*‐TKD mutations were included into this retrospective analysis, with a median of six patients per centre (Figure S1). Median age was 58.5 years (range 21–84). The cohort comprised 57% female and 43% male patients. The majority of patients (72.5%) had a good performance status (Karnofsky index ≥80%). Most patients were diagnosed with de novo AML (81.4%). At diagnosis, 36.5% were classified as adverse‐risk, 43.6% as intermediate‐risk and 19.9% as favourable‐risk. Complex cytogenetic abnormalities were present in 6.4% of patients. The majority harboured *FLT3*‐ITD mutations (80.8%), while 14.1% had *FLT3*‐TKD mutations. Eight patients (5.1%) carried both mutation types. Baseline patient and disease characteristics including treatment prior to gilteritinib are summarized in Table 1.

**TABLE 1:** Patients' baseline characteristics.

	*FLT3* ^mut^ patients with gilteritinib treatment (*n* = 156)
Age (years), median (range)	58.5 (21–84)
Sex, *n*/*N* (%)	
Female	89/156 (57.1)
Male	67/156 (42.9)
Karnofsky performance status, *n*/*N* (%)	
≥80%	108/149 (72.5)
<80%	41/149 (27.5)
Miss	7
Disease status, *n*/*N* (%)	
De novo	127/156 (81.4)
s‐AML	20/156 (12.8)
t‐AML	9/156 (5.8)
Complex karyotype, *n*/*N* (%)	10/156 (6.4)
ELN2017 risk, *n*/*N* (%)	
Favourable	31/156 (19.9)
Intermediate	68/156 (43.6)
Adverse	57/156 (36.5)
*FLT3* mutation distribution	
*FLT3*‐ITD	126/156 (80.8)
*FLT3*‐TKD	22/156 (14.1)
*FLT3*‐ITD + *FLT3*‐TKD	8/156 (5.1)
Induction therapy (IT), *n*/*N* (%)	
7 + 3	109/156 (69.9)
Vyxeos	10/156 (6.4)
HAM	5/156 (3.2)
Other	14/156 (9)
+midostaurin	61/138 (44.2)
HMA ± venetoclax	17/156 (10.9)
Hydroxyurea (hydroxycarbamide)	1/156 (0.6)
Remission status after IT, *n*/*N* (%)	
CR/CRi	86/152 (56.6)
Partial remission	3/152 (2)
Progress/refractory	63/152 (41.4)
Unknown	4
Previous treatment lines	
1	124/156 (79.5)
≥2	32/156 (20.5)
Number of FLT3i prior gilteritinib, *n*/*N* (%)	
0	84/156 (53.8)
1	54/156 (34.6)
2	15/156 (9.6)
3	3/156 (2)

Abbreviations: alloHSCT, allogeneic haematopoietic stem cell transplantation; AML, acute myeloid leukaemia; CR, complete remission; CRi, incomplete remission; ELN, European Leukemia Net; FLT3i, FLT3 inhibitor; FLT3‐ITD, FLT3‐ internal tandem duplication; *FLT3* ^mut^, FMS‐like tyrosine kinase 3 mutation; FLT3‐TKD, FLT3‐tyrosine kinase domain; HAM, high‐dose cytarabine + mitoxantrone; HMA, hypomethylating agent; IT, induction therapy; s‐AML, secondary AML; t‐AML, therapy‐related AML.

### Previous treatment

Following initial AML diagnosis, intensive IT was administered in 88.5% of patients, whereas 11.5% were considered unfit for intensive treatment and mainly received hypomethylating agents. Among the intensively treated patients, 44.2% received FLT3i midostaurin as part of frontline therapy. After IT, CR/incomplete remission (CRi) was achieved in 56.6% of patients, while 41.4% had progressive or refractory disease or achieved partial remission (2%), prompting salvage treatment (Table 1). Overall, 55.1% (*n* = 86) of patients had undergone alloHSCT prior to relapse or refractory disease and subsequent gilteritinib treatment. Most patients (79.5%) received only one previous treatment line before gilteritinib salvage, while 20.5% underwent at least two prior therapy lines. Eighty‐four patients (53.8%) received gilteritinib as their first FLT3i, whereas 72 patients (46.2%) had prior exposure to at least 1 FLT3i (Table 1).

### Gilteritinib treatment sequence

Overall, 44% (*n* = 69) had primary refractory disease and 56% (*n* = 87) relapsed after initial response, qualifying for gilteritinib treatment. Among the 156 *FLT3* ^mut^ r/r AML patients, gilteritinib was administered in three main therapeutic sequences (Figure 1; Figure S2).

**FIGURE 1:**
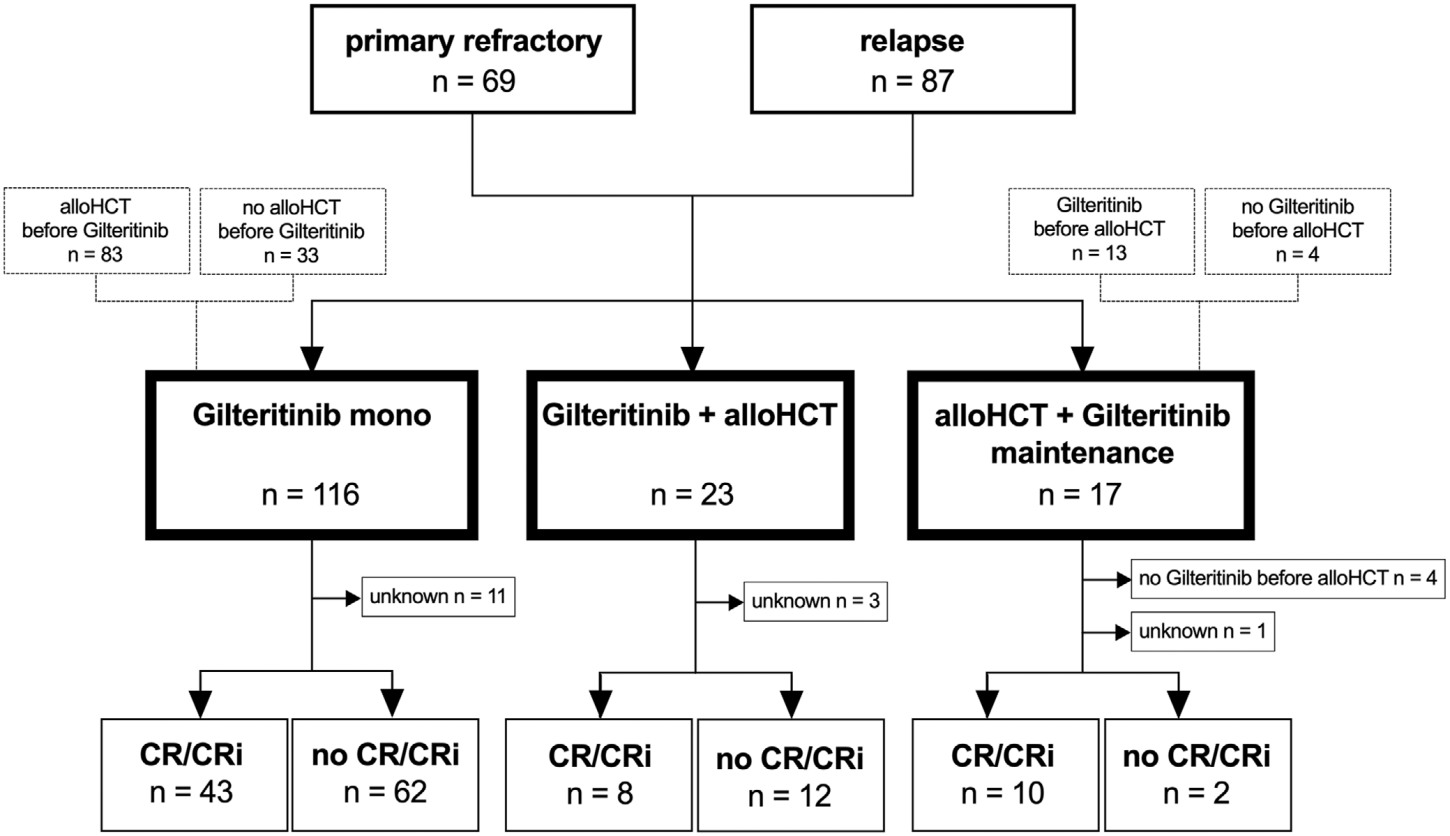
CONSORT diagram of patient distributions. CONSORT diagram illustrating the distribution of study patients according to disease status qualifying for gilteritinib treatment, the respective sequential therapies and the best response achieved. Boxes represent patient subgroups; arrows indicate therapy sequence. alloHSCT, allogeneic haematopoietic stem cell transplantation; CR/CRi, complete remission/incomplete remission.

#### Group 1: Gilteritinib monotherapy

The majority of *FLT3* ^mut^ r/r AML patients (74.4%, *n* = 116) received gilteritinib as single‐agent therapy (‘gilteritinib mono’ group). Of these, 83 patients (71.6%) had previously undergone alloHSCT.

#### Group 2: Gilteritinib followed by alloHSCT

Fourty patients (25.6%) underwent first (*n* = 37, 93%) or second (*n* = 3, 7%) alloHSCT after gilteritinib salvage treatment. Most of these patients (*n* = 23, 58%) received gilteritinib followed by alloHSCT with no further gilteritinib maintenance (‘gilteritinib + alloHSCT’ group). Eight patients received sorafenib maintenance after alloHSCT (Figure S2), while 15 patients did not receive any maintenance therapy (‘gilteritinib + alloHSCT without maintenance’ group).

#### Group 3: Gilteritinib + alloHSCT + gilteritinib maintenance

Seventeen patients (42.5% of alloHSCT patients) resumed gilteritinib as maintenance therapy following alloHSCT. Four of these patients had not received gilteritinib prior to alloHSCT. The remaining 13 patients constitute the ‘gilteritinib + alloHSCT + gilteritinib maintenance’ group.

### Gilteritinib treatment

Median duration of gilteritinib treatment was 2.5 months (range 0.07–14.7) in patients without subsequent alloHSCT, 2.35 months (range 0.66–30.83) when used as bridge to alloHSCT, and 16.56 months (range 1.87–28.1) in the maintenance setting. Median time from alloHSCT to initiation of gilteritinib maintenance was 2 months (range 0.89–9.83).

The most common reasons for discontinuation of gilteritinib were relapse or disease progression (38.4%) and alloHSCT (11.3%). Frequent adverse events associated with gilteritinib were hepatic toxicity (6.4%) and cardiac events (4.4%), followed by cytopenia and infections/sepsis, myalgia, nausea and vertigo. Overall tolerability was favourable with 57.1% of patients discontinuing treatment for reasons other than toxicity. Patient characteristics during gilteritinib treatment are summarized in Table 2.

**TABLE 2:** Gilteritinib treatment characteristics.

	*FLT3* ^mut^ patients with gilteritinib treatment (*n* = 156)
Indication for gilteritinib, *n*/*N* (%)	
Primary refractory	69/156 (44.2)
Relapse	87/156 (55.8)
Gilteritinib treatment, *n*/*N* (%)	
Before alloHSCT	36/156 (23.1)
As maintenance after alloHSCT	17/156 (10.9)
Without alloHSCT	116/156 (74.4)
Duration of gilteritinib treatment (months), median (range)	
Before alloHSCT	2.35 (0.66–30.83)
As maintenance after alloHSCT	16.56 (1.87–28.1)
Without alloHSCT	2.51 (0.07–14.7)
Best response to gilteritinib treatment, *n*/*N* (%)	
CR/CRi	63/139 (45.3)
Progress/refractory	39/139 (28.1)
Partial response	37/139 (26.6)
Unknown	17
EOT for gilteritinib treatment	
Relapse/progress	58/151 (38.4)
None	44/151 (29.1)
alloHSCT	17/151 (11.3)
Other	15/151 (9.9)
AE	9/151 (6)
Death	8/151 (5.3)
Unknown	5
AESIs during gilteritinib treatment	
Hepatic toxicity	10/156 (6.4)
Prolonged QTc interval	6/156 (3.8)
Cardiac toxicity	1/156 (0.6)
Gastrointestinal bleeding	1/156 (0.6)
Other	49/156 (31.4)
None	89/156 (57.1)

Abbreviations: AE, adverse event; AESI, adverse event of special interest; alloHSCT, allogeneic haematopoietic stem cell transplantation; CR, complete remission; CRi, incomplete remission; EOT, end of treatment; *FLT3* ^mut^, FMS‐like tyrosine kinase 3 mutation; QTc, corrected QT interval.

### Response to gilteritinib treatment

Across the entire cohort, the best response achieved at any time during gilteritinib treatment was CR/CRi in 45.3% of patients, while 54.7% did not achieve CR/CRi (Figure 1; Table 2). The number of prior treatment lines did not affect response to gilteritinib (*p* = 0.858; Table S2). Similarly, prior exposure to FLT3i resulted in comparable CR/CRi rates (48.4% vs. 42.9% in FLT3i‐naïve patients; *p* = 0.095). Although *FLT3*‐TKD^mut^ patients showed lower CR/CRi rates, no statistically significant difference was observed among mutation subtypes (*p* = 0.98). When comparing therapeutic strategies, significantly higher CR/CRi rates were observed in patients receiving gilteritinib combined with alloHSCT and gilteritinib maintenance (41% vs. 40% vs. 83%; *p* = 0.02; Table 3A). No significant differences in pre‐transplant disease response were identified between alloHSCT patients with or without subsequent gilteritinib maintenance (Table 3B).

**TABLE 3:** Remission status.

(A) Best remission after gilteritinib and sequential treatment				
Therapeutic sequence	CR/CRi	no CR	*p*‐value	
Gilteritinib mono, *n*/*N* (%)	43/105 (41)	62/105 (59)		**0.02**
Gilteritinib + alloHSCT, *n*/*N* (%)	8/20 (40)	12/20 (60)	**0.02**	
Gilteritinib + alloHSCT + gilteritinib maintenance, *n*/*N* (%)	10/12 (83)	2/12 (17)		

(B) Remission status after gilteritinib treatment before alloHSCT			
Therapeutic sequence	CR/CRi	Partial remission	Relapse/refractory
Gilteritinib + alloHSCT, *n*/*N* (%)	7/11 (63.6)	3/11 (27.3)	1/11 (9.1)
Gilteritinib + alloHSCT + gilteritinib maintenance, *n*/*N* (%)	6/7 (85.7)	1/7 (14.3)	0/7 (0)
***p*‐value**	0.31	0.52	0.41

*Note*: *p*‐values were calculated with Pearson's chi‐squared test, significant results are highlighted in bold.

Abbreviations: alloHSCT, allogeneic haematopoietic stem cell transplantation; CR, complete remission; CRi, incomplete remission.

### Survival and impact of therapeutic sequences including alloHSCT

With a median follow‐up of 13.1 months (95% confidence interval [CI] 10.6–16.3), median OS for the entire cohort was 10.0 months (95% CI 8–not reached). One‐year OS was 49% (95% CI 40–59). Neither the number of prior treatment lines nor prior FLT3i exposure influenced survival (Table S2). *FLT3*‐ITD^mut^ patients showed better survival under gilteritinib treatment (median OS 13 months; 1‐year OS 51%) compared to *FLT3*‐TKD^mut^ patients (median OS 7.8 months; 1‐year OS 39%) or double mutated patients (median OS 3.8 months; 1‐year OS 30%). However, those differences were not statistically significant (*p* = 0.3; Table S2).

Patients receiving gilteritinib without subsequent alloHSCT had significantly shorter median OS of 7.7 months (95% CI 6.4–9.7) and a 1‐year OS of 34% (95% CI 25–47), compared with treatment strategies including alloHSCT. Median OS was not reached in the gilteritinib + alloHSCT group (HR = 0.28, 95% CI 0.11–0.71, *p* = 0.007) or in the alloHSCT + gilteritinib maintenance group (HR = 0.06, 95% CI 0.01–0.46, *p* = 0.006). One‐year OS from alloHSCT was 76% (95% CI 60–97), and 1‐year OS from start of maintenance therapy was 92% (95% CI 79–100). Transplant‐related variables including HCT‐CI score, donor type, conditioning intensity and use of donor lymphocyte infusion (DLI) did not differ significantly between alloHSCT groups (Table 4).

**TABLE 4:** Patients’ characteristics and survival according to gilteritinib treatment sequences.

	*FLT3* ^mut^ patients with gilteritinib treatment (*n* = 156)	*p*‐value
Overall survival with gilteritinib (months), median (range)		
Gilteritinib mono	7.7 (6.4–9.7)	**<0.001**
Gilteritinib + alloHSCT	*not reached*	
alloHSCT + gilteritinib maintenance	*not reached*	
1‐year overall survival with gilteritinib (%), 95% CI (%)		
Gilteritinib mono	34 (25–47)	**<0.001**
Gilteritinib + alloHSCT	76 (60–97)	
alloHSCT + gilteritinib maintenance	92 (79–100)	
HCT‐CI at time of alloHSCT, *n*/*N* (%)		
Gilteritinib + alloHSCT		0.33
0	9/23 (39.1)	
1–2	5/23 (21.7)	
≥3	6/23 (26.1)	
Unknown	3/23 (13)	
alloHSCT + gilteritinib maintenance		
0	8/17 (47.1)	
1–2	1/17 (5.9)	
≥3	3/17 (17.6)	
Unknown	5/17 (29.4)	
Conditioning, *n*/*N* (%)		
Gilteritinib + alloHSCT		**0.04**
NMA	1/23 (4.3)	
RIC	10/23 (43.5)	
MAC	8/23 (34.8)	
Sequential	4/23 (17.4)	
alloHSCT + gilteritinib maintenance		
RIC	7/17 (41.2)	
MAC	6/17 (35.3)	
Sequential	4/17 (23.5)	
Donor type, *n*/*N* (%)		
Gilteritinib + alloHSCT		0.299
MSD	1/23 (4.3)	
MUD	19/23 (82.6)	
Haplo	3/23 (13.1)	
alloHSCT + Gilteritinib maintenance		
MSD	1/17 (5.9)	
MUD	16/17 (94.1)	
Haplo	0/17 (0)	
DLI after alloHSCT, *n*/*N* (%)		
Gilteritinib mono		0.66
Yes	18/116 (15.5)	
No/unknown	98/116 (84.5)	
Gilteritinib + alloHSCT		
Yes	2/23 (8.7)	
No/unknown	21/23 (91.3)	
alloHSCT + gilteritinib maintenance		
Yes	2/17 (11.8)	
No/unknown	15/17 (88.2)	

*Note*: *p*‐values were calculated with Pearson's chi‐squared test, significant results are highlighted in bold.

Abbreviations: 95% CI, 95% confidence interval; alloHSCT, allogeneic haematopoietic stem cell transplantation; DLI, donor lymphocyte infusion; *FLT3* ^mut^, FMS‐like tyrosine kinase 3 mutation; Haplo, haploidentical; HCT‐CI, hematopoietic stem cell transplantation‐specific comorbidity index; MAC, myeloablative conditioning; MSD, matched sibling donor; MUD, matched unrelated donor; NMA, non‐myeloablative; RIC, reduced intensity conditioning.

Further subgroup analyses demonstrated improved OS when alloHSCT was performed as part of gilteritinib salvage (alloHSCT as time‐dependent covariate; HR = 3.6, 95% CI 1.6–7.9, *p* < 0.01; Figure 2A). Patients of the gilteritinib mono cohort with prior alloHSCT (*n* = 83, ‘gilteritinib mono, previous alloHSCT’) showed a trend towards improved OS compared to those without previous transplantation (*n* = 33, ‘gilteritinib mono, no previous alloHSCT’; HR = 1.6, 95% CI 0.9–2.7, *p* = 0.1; Figure 2B), with 1‐year OS probability of 38% (95% CI 28–53) vs. 22% (95% CI 9.4–51). Superior survival was achieved if gilteritinib was resumed after alloHSCT compared to no maintenance (1‐year OS 92%, 95% CI 79–100 vs. 58%, 95% CI 36–95; Figure 2C,D).

**FIGURE 2:**
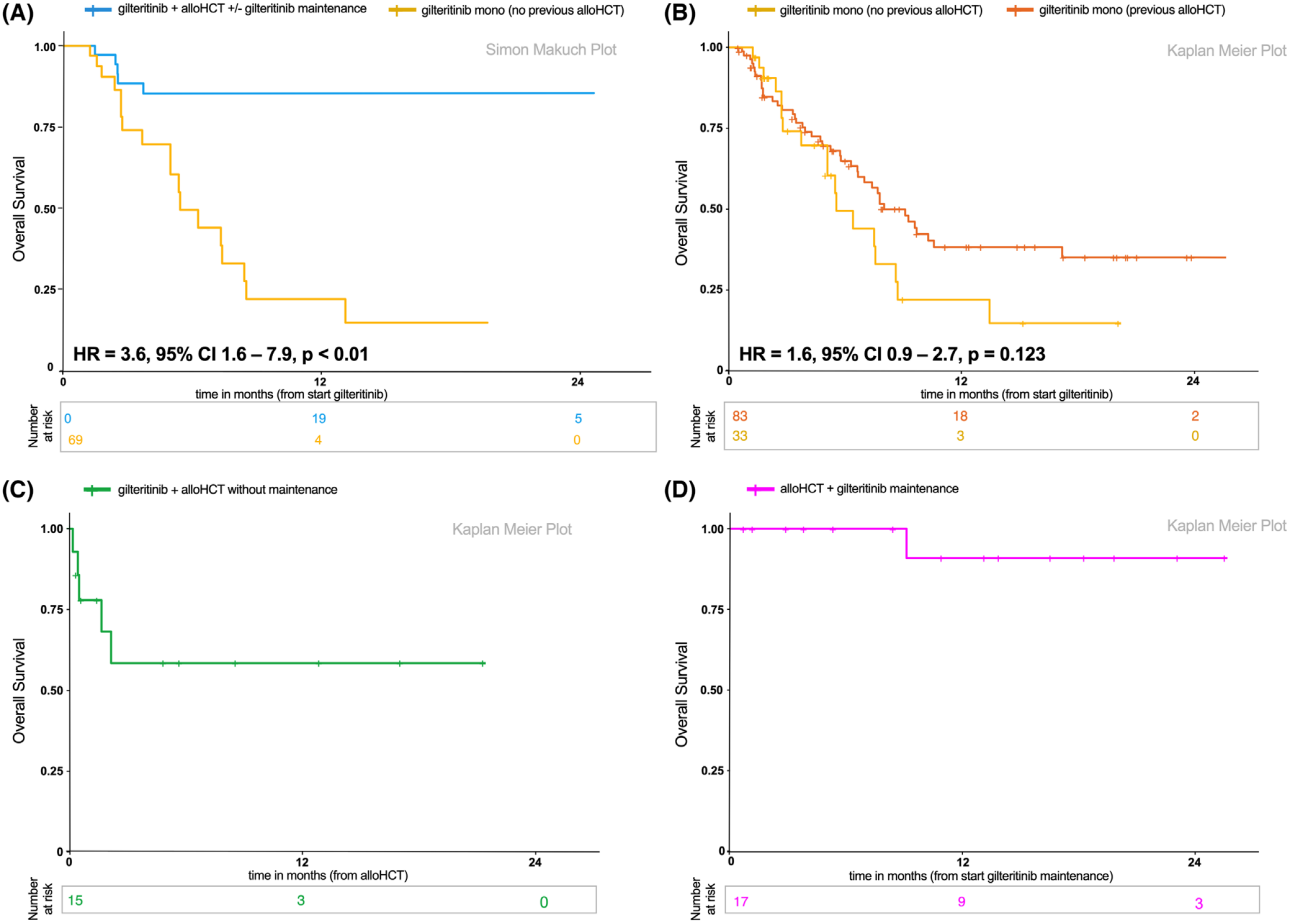
Overall survival according to therapeutic sequences of gilteritinib Overall survival of: (A) gilteritinib followed by allogeneic haematopoietic stem cell transplantation (alloHSCT) (blue) versus gilteritinib monotherapy without prior alloHSCT (yellow); (B) gilteritinib monotherapy without prior alloHSCT (yellow) versus gilteritinib after relapse following alloHSCT (orange); (C) gilteritinib followed by immediate alloHSCT without gilteritinib maintenance (green); (D) gilteritinib maintenance after alloHSCT (pink). *p*‐values were calculated using a Cox model with time‐dependent modelling of alloHSCT for Simon–Makuch plots. Time is shown in months. CI, confidence interval; HR, hazard ratio.

### Maintenance after alloHSCT

Seventeen patients resumed gilteritinib after alloHSCT and eight patients received sorafenib maintenance. Median time to initiation of maintenance was comparable (sorafenib: 2.37 months, range 1.38–19.8; gilteritinib: 2 months, range 0.89–9.8). Both maintenance strategies were associated with excellent outcomes, with 1‐year OS rates of 100% (sorafenib) and 91% (gilteritinib) respectively (*p* = 0.4; Figure S3).

### Impact of FLT3 mutation subtype

*FLT3* ^mut^ subtype did not significantly affect survival when patients received gilteritinib without subsequent alloHSCT (*p* = 0.91). In the alloHSCT setting, *FLT3*‐ITD^mut^ patients showed favourable outcomes compared to *FLT3*‐TKD^mut^ patients, particularly when gilteritinib maintenance was administered (*FLT3‐*ITD: 1‐year OS 92%, 95% CI 77–100) and *FLT3*‐TKD: 1‐year OS 100%; for both median OS was not reached, although subgroup size was limited (Figure 3A–C).

**FIGURE 3:**
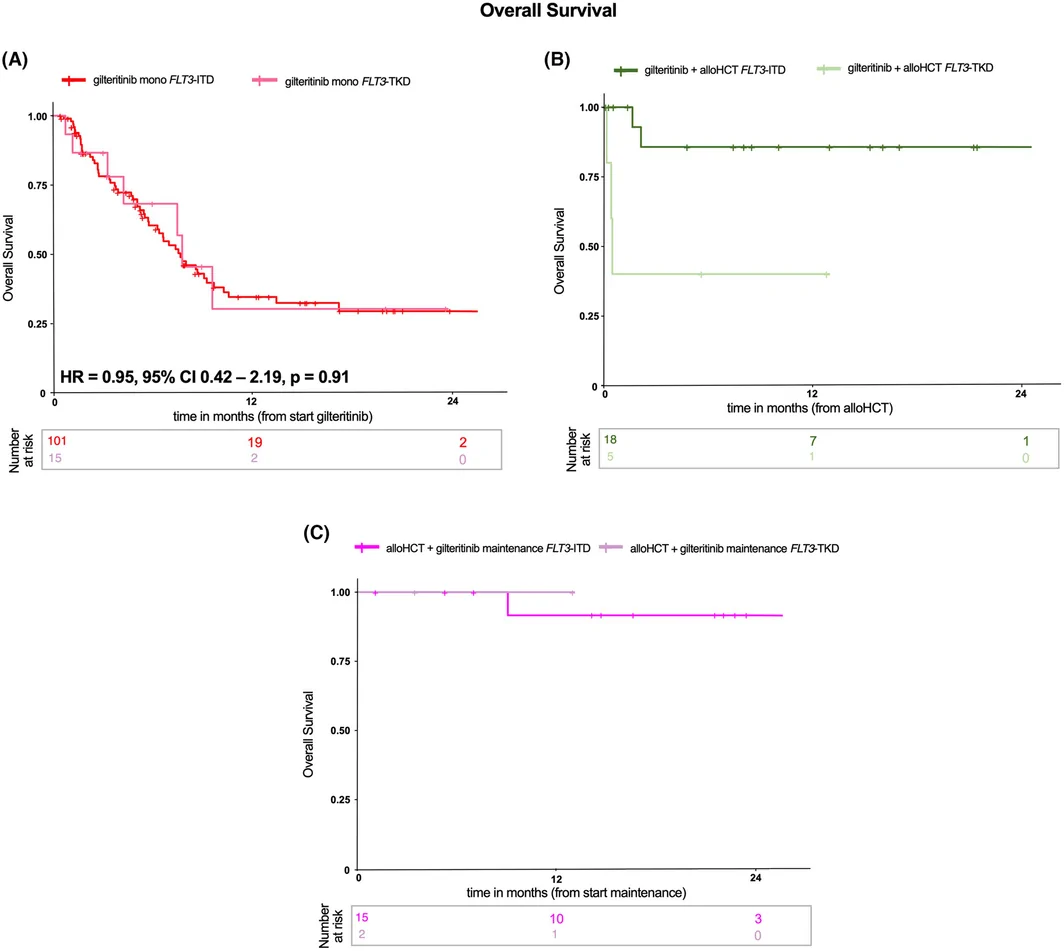
Overall survival according to FLT3‐ITD and FLT3‐TKD mutation subtype. Overall survival plots stratified by FLT3 mutation subtype: (A) gilteritinib monotherapy: FLT3‐ITD (red) versus FLT3‐TKD (light red); (B) gilteritinib followed by allogeneic haematopoietic stem cell transplantation (alloHSCT): FLT3‐ITD (dark green) versus FLT3‐TKD (light green); (C) gilteritinib maintenance after alloHSCT: FLT3‐ITD (pink) versus FLT3‐TKD (purple). *p*‐values were determined using a Cox model. Time is shown in months. CI, confidence interval; FLT3, FMS‐like tyrosine kinase 3; FLT3‐ITD, FLT3‐are internal tandem duplication; FLT3‐TKD, FLT3‐tyrosine kinase domain; HR, hazard ratio.

### Donor lymphocyte infusions and gilteritinib

DLIs were administered in conjunction with gilteritinib treatment in 18 patients in the gilteritinib monotherapy group and two patients each in the gilteritinib + alloHSCT group and in the gilteritinib + alloHSCT + gilteritinib maintenance group (Table 4). The mean time from initiation of gilteritinib to first DLI administration was 109, 278, and 287 days respectively. One‐year OS calculated from the first DLI was 52% (95% CI 31–88) in the gilteritinib monotherapy group and 100% in both alloHSCT‐based treatment groups.

## DISCUSSION

Our cohort of 156 patients with r/r *FLT3* ^mut^ AML treated with gilteritinib single‐agent adds one of the largest published data collections to the growing body of evidence on the real‐world efficacy of gilteritinib and demonstrates clinically meaningful activity. The observed response rates and survival outcomes are consistent with, and in part exceed, those reported in randomized controlled trials and other real‐world analyses. In our cohort, patients without subsequent alloHSCT achieved CR/CRi rates of 41%. This is in line with the COMMODORE trial and two large real‐world trials by the British National Cancer Research Institute and the French Innovative Leukemia Organization/Acute Leukemia French Association groups, whereas slightly higher responses around 50% were reported in the ADMIRAL trial and real‐world experiences from Israel and Italy.^33^, ^34^, ^47^, ^48^, ^49^, ^50^, ^51^

Importantly, our data confirm that initial response to gilteritinib does not necessarily translate into long‐term survival unless consolidated by alloHSCT. Nevertheless, the overall CR/CRi rate of 45.3% in our cohort was associated with median OS of 10.0 months, which ranges among the longest published survival both across the ADMIRAL (9.3 months) and COMMODORE (9.6 months) trial and across real‐world data. Several factors may have contributed to this favourable outcome, including the relatively young median age of our cohort and a high proportion of patients considered eligible for alloHSCT. In contrast, cohorts with older patient populations and reduced performance status, such as the French real‐world cohort, reported inferior outcomes, highlighting the importance of patient fitness for long‐term disease control.

Eligibility for alloHSCT remains one of the most critical determinants of long‐term survival in r/r AML. In our cohort, approximately 25% of patients underwent alloHSCT after gilteritinib salvage, representing one of the highest transplant rates reported in real‐world studies and comparable to the cohort described by Othman et al., who reported a similar median age, transplant rate and median OS of 9.5 months.^47^ Notably, gilteritinib maintenance after alloHSCT was associated with the highest CR/CRi rates and best survival, although disease response prior to alloHSCT did not differ between patients receiving maintenance and those who did not. These findings are consistent with post‐hoc analyses of the ADMIRAL trial, which also demonstrated improved survival in patients continuing gilteritinib after transplantation. It is important to acknowledge that retrospective analyses of maintenance strategies inherently are biased towards a more favourable patient subgroup, as only those able to initiate maintenance are captured. Nevertheless, the consistently observed association between post‐alloHSCT FLT3 inhibition and improved survival across different cohorts supports the concept of maintenance therapy as an integral component of *FLT3* ^mut^ AML management.

Our cohort is characterized by a high proportion of patients relapsing after prior alloHSCT, likely reflecting the tertiary care setting of participating university hospitals. Intriguingly, both response rates and survival were superior in these patients compared with patients without prior alloHSCT. This observation may primarily reflect younger age and better performance status, but may also suggest a contributory graft‐versus‐leukaemia (GvL) effect enhancing the efficacy of gilteritinib in the presence of donor‐derived immune surveillance.^52^ Furthermore, the role of DLI as part of gilteritinib‐based salvage strategies, and particularly the optimal timing of their administration, remains to be defined. Future prospective studies should investigate potential synergistic effects of DLI and gilteritinib in augmenting GvL activity, while also balancing the increased risk of graft‐versus‐host disease associated with DLI. Although limited by the retrospective design of this study, our findings suggest that the addition of DLI to gilteritinib may further improve OS in patients relapsing after alloHSCT.

Based on our results, gilteritinib should be strongly considered as a bridge‐to‐transplant strategy in eligible patients and alloHSCT should be pursued as soon as possible if therapy intensification is feasible. Furthermore, our data support the early resumption of gilteritinib as maintenance therapy after alloHSCT whenever clinically possible. These findings align with published data by Terao et al., who demonstrated improved outcomes with early initiation of post‐transplant gilteritinib maintenance.^53^ However, in the era of refined molecular diagnostics including measurable residual disease monitoring, important questions remain regarding optimal patient selection, timing and duration of maintenance therapy.^41^, ^54^

In our cohort, three patients were allografted twice during the course of their disease and subsequently received gilteritinib maintenance, with survival outcomes exceeding those typically reported for second alloHSCT in AML.^55^ These observations further support the rationale for integrating FLT3i into sequential treatment strategies, particularly given the diverse primary and secondary mechanisms of resistance to FLT3i that limit long‐term disease control with monotherapy.^56^

Consistent with previous studies, our results suggest a higher efficacy of gilteritinib in patients harbouring *FLT3*‐ITD mutations compared to *FLT3*‐TKD mutations, particularly in the context of alloHSCT. Importantly, pretreatment with first‐generation FLT3i, documented in nearly half of our cohort, did not adversely affect response or survival, supporting the use of gilteritinib regardless of prior FLT3i treatment history.

While our study represents one of the largest real‐world analyses of gilteritinib that demonstrates favourable survival outcomes, several limitations must be acknowledged. The retrospective design is inherently associated with selection bias, although we attempted to minimize this by requiring documentation of all consecutively treated patients at participating centres. In addition, incomplete pre‐transplant bone marrow assessments limited the ability to uniformly capture remission status before alloHSCT, as several patients proceeded directly to conditioning. Despite the overall cohort size, subgroup analyses were limited by small patient numbers, restricting the power of multivariable analyses.

In conclusion, this large multicentre real‐world analysis demonstrates that gilteritinib induces remissions in the majority of patients with r/r *FLT3* ^mut^ AML. Durable survival is primarily observed in patients undergoing consolidative alloHSCT and continued gilteritinib therapy after transplantation. Future prospective studies are warranted to further define optimal sequencing strategies and to explore combination approaches with other targeted agents to further improve long‐term outcomes in this high‐risk patient population.

## AUTHOR CONTRIBUTIONS

**Desiree Kunadt:** Conceptualization; investigation; writing – original draft; methodology; validation; writing – review and editing; formal analysis; project administration; data curation; resources; visualization. **Johannes Schetelig:** Conceptualization; investigation; writing – review and editing; methodology; validation; formal analysis; supervision; resources. **Martin Bornhäuser:** Resources; writing – review and editing. **Christian Thiede:** Resources. **Jan M. Middeke:** Conceptualization; investigation; writing – review and editing; data curation; resources; supervision; formal analysis; project administration. **Friedrich Stölzel:** Data curation; supervision; resources; formal analysis; writing – review and editing; conceptualization; investigation; project administration. **Wolfgang Bethge:** Writing – review and editing; data curation; resources. **Christoph Schliemann:** Writing – review and editing; data curation; resources. **Björn Steffen:** Writing – review and editing; data curation; resources. **Eva Wagner‐Drouet:** Writing – review and editing; resources; data curation. **Matthias Stelljes:** Writing – review and editing; data curation; resources. **Jan Frederic Weller:** Writing – review and editing; data curation; resources. **Sven Zukunft:** Methodology; validation; writing – review and editing; formal analysis; data curation; software; visualization. **Oliver Kriege:** Writing – review and editing; data curation; resources. **Christoph Röllig:** Conceptualization; investigation; writing – review and editing; formal analysis; data curation; resources; supervision. **Lars Fransecky:** Writing – review and editing; data curation; resources. **Julian Ronnacker:** Writing – review and editing; data curation; resources. **Elisa Sala:** Data curation; resources; writing – review and editing. **Stephan Metzelder:** Writing – review and editing; resources; data curation. **Gesine Bug:** Writing – review and editing; data curation; resources. **Andreas Hochhaus:** Writing – review and editing; resources. **Klaus Metzeler:** Writing – review and editing; data curation; resources. **Uwe Platzbecker:** Writing – review and editing; data curation; resources. **Sabine Kayser:** Writing – review and editing; data curation; resources. **Khouloud Kouidri:** Data curation; resources; writing – review and editing. **Tim H. Brümmendorf:** Writing – review and editing; data curation; resources. **Edgar Jost:** Writing – review and editing; data curation; resources. **Claudia Baldus:** Writing – review and editing; data curation; resources. **Thomas Schroeder:** Writing – review and editing; data curation; resources. **Andreas Burchert:** Writing – review and editing; resources; data curation. **Lambros Kordelas:** Writing – review and editing; data curation; resources. **Inken Hilgendorf:** Writing – review and editing; data curation; resources. **Johanna Tischer:** Writing – review and editing; data curation; resources. **Tobias Herold:** Writing – review and editing; data curation; resources. **David Nachbaur:** Writing – review and editing; data curation; resources. **Anna Dorottya Doleschall:** Writing – review and editing; data curation; resources. **Udo Holtick:** Writing – review and editing; data curation; resources. **Guido Kobbe:** Writing – review and editing; data curation; resources. **Elena Stauffer:** Writing – review and editing; data curation; resources. **Julia M. Unglaub:** Writing – review and editing; data curation; resources. **Tim Sauer:** Writing – review and editing; data curation; resources. **Sabrina Kraus:** Writing – review and editing; data curation; resources. **Jochen J. Frietsch:** Writing – review and editing; data curation; resources. **William Krüger:** Resources; data curation; writing – review and editing. **Mathias Hänel:** Resources. **Normann Steiner:** Writing – review and editing; data curation; resources. **Nicolaus Kröger:** Resources. **Laura Reinhardt:** Writing – review and editing; data curation; resources. **Judith Schaffrath:** Writing – review and editing; data curation; resources. **Jennifer Kaivers:** Writing – review and editing; data curation; resources. **Markus Maulhardt:** Writing – review and editing; data curation; resources. **Paul Sebastian Jäger:** Writing – review and editing; data curation; resources. **Hermann Einsele:** Resources. **Anne Brandenburg:** Writing – review and editing; data curation; resources. **Tobias A. W. Holderried:** Writing – review and editing; data curation; resources.

## CONFLICT OF INTEREST STATEMENT

The authors have no conflicts of interest to disclose.

## ETHICS STATEMENT

This study included patients enrolled in the Study Alliance Leukemia (SAL) Registry (NCT03188874) and the German Cooperative Transplant Study Group. All analyses were approved by the respective institutional ethics committees and conducted in accordance with the Declaration of Helsinki.

## PATIENT CONSENT STATEMENT

This study included patients enrolled in the Study Alliance Leukemia (SAL) Registry (NCT03188874) and the German Cooperative Transplant Study Group. All patients provided their written informed consent including analysis of data.

## CLINICAL TRIAL REGISTRATION

Patients were enrolled in the Study Alliance Leukemia (SAL) Registry (NCT03188874) and the German Cooperative Transplant Study Group.

## Supporting information


**Figure S1.** Patients contributed per centre.

**Figure S2.** Schematic overview of therapeutic subgroups.

**Figure S3.** Overall survival and progression‐free survival of sorafenib versus gilteritinib maintenance.

**Table S1.** Conditioning protocols.

**Table S2.** Impact of treatment prior gilteritinib and *FLT3* mutation type on outcome.

## Data Availability

Data are available from the corresponding author upon request.
