# Supplementary material for: Treatment sequence with gilteritinib and allogeneic haematopoietic stem cell transplantation in relapsed/refractory FMS‐like tyrosine kinase 3 (FLT3)‐mutated acute myeloid leukaemia patients: A multicentre real‐world study
Source: Br J Haematol. 2026 Aug 18;209(4):1783–96. doi: 10.1111/bjh.70735 (PMC13674134; doi:10.1111/bjh.70735)
Supplement: Supplementary file 1 [file BJH-209-1783-s001.docx]

**SUPPLEMENT**

**Supplemental Table S01 Conditioning protocols**

| **NMA** | **RIC** | **MAC** | **Sequential** |
| --- | --- | --- | --- |
| Flu/TBI 2 Gy | Flu/Treo | Flu/Bu4 | FLAMSA-RIC |
|  | Flu/Bu2 | Bu/Cy | high dose Mel - RIC |
|  | Flu/Mel | protocols with TBI > 8 Gy,  e.g. Cy/TBI 12 Gy |  |
|  | protocols with TBI ≤ 8Gy, e.g. Flu/TBI 8Gy |  |  |

**Supplemental Table 1** Conditioning protocols according to intensity

*Abbreviations:* Bu, busulfan; Cy, cyclophosphamide; FLAMSA, fludarabine and amsacrine and cytarabine; Flu, fludarabine; Gy, Gray; MAC, myeloablative conditioning; Mel, melphalan; NMA, nonmyeloablative conditioning; RIC, reduced intensity conditioning; TBI, total body irradiation,
