# Supplementary material for: Treatment sequence with gilteritinib and allogeneic haematopoietic stem cell transplantation in relapsed/refractory FMS‐like tyrosine kinase 3 (FLT3)‐mutated acute myeloid leukaemia patients: A multicentre real‐world study
Source: Br J Haematol. 2026 Aug 18;209(4):1783–96. doi: 10.1111/bjh.70735 (PMC13674134; doi:10.1111/bjh.70735)
Supplement: Supplementary file 2 [file BJH-209-1783-s002.docx]

**SUPPLEMENT**

**Supplemental Table S02 Impact of treatment prior gilteritinib and *FLT3* mutation type on outcome**

|  | **Total, n/N (%)** | **CR/CRi, n/N (%)** | ***p-value*** | **Median OS, months (95% CI)** | **1-year OS, % (95% CI)** | ***p-value*** |
| --- | --- | --- | --- | --- | --- | --- |
| **Previous treatment lines**  **1**  **≥ 2**  **missing (n)** | 124/156 (79.5)  32/156 (20.5)  - | 51/110 (46.4)  12/29 (41.4)  17 | .858 | 11 (8.6 – not reached)  10 (5.1 – not reached)  - | 49 (40 – 61)  46 (29 – 71)  - | .7 |
| **Previous FLT3i treatment**  **no**  **yes**  **missing (n)** | 84/156 (53.8)  72/156 (46.2)  - | 33/77 (42.9)  30/62 (48.4)  17 | .095 | 9.1 (7.4 – not reached)  not reached (9.1 – not reached)  - | 43 (32 – 57)  55 (44 – 70)  - | .3 |
| ***FLT3 status***  ***FLT3-ITD***  ***FLT3-TKD***  ***FLT3-ITD* + *FLT3-TKD*** | 126/156 (80.8)  22/156 (14.1)  8/156 (5.1) | 54/116 (46.6)  6/21 (28.6)  3/8 (37.5) | .98 | 13.0 (8.7 – not reached)  7.8 (4.4 – not reached)  3.8 (3.5 – not reached) | 51 (42 – 62)  39 (20 – 76)  30 (9.5 – 94) | .3 |

*p-values for categorical variables were calculated with Pearson’s Chi-squared test, ANOVA F‑test from a linear model used to compare group means for continuous variables, significant results are highlighted in bold.*
